# Necrotizing fasciitis – a diagnostic dilemma: two case reports

**DOI:** 10.1186/1752-1947-8-229

**Published:** 2014-06-25

**Authors:** Mitrakrishnan Rayno Navinan, Jevon Yudhishdran, Thambyaiah Kandeepan, Aruna Kulatunga

**Affiliations:** 1 National Hospital of Sri Lanka, Regent Street, Colombo 10, Sri Lanka

**Keywords:** Necrotizing fasciitis, Necrotizing soft tissue infections, Systemic lupus erythematosus

## Abstract

**Introduction:**

Necrotizing soft tissue infections can affect various tissue planes. Although predisposing etiologies are many, they mostly center on impaired immunity occurring directly or indirectly and loss of integrity of protective barriers which predispose to infection. The nonspecific presentation may delay diagnosis and favor high mortality.

**Case presentation:**

Two case vignettes are presented. The first patient, a 44-year-old healthy South Asian man with a history of repeated minor traumatic injury presented to a primary health care center with a swollen left lower limb. He was treated with antibiotics with an initial diagnosis of cellulitis. Because he deteriorated rapidly and additionally developed intestinal obstruction, he was transferred to our hospital which is a tertiary health care center for further evaluation and management. Prompt clinical diagnosis of necrotizing soft tissue infection was made and confirmed on magnetic resonance imaging as necrotizing fasciitis. Urgent debridement was done, but the already spread infection resulted in rapid clinical deterioration with resultant mortality. The second patient was a 35-year-old South Asian woman with systemic lupus erythematous receiving immunosuppressive therapy who developed left lower limb pain and fever. Medical attention was sought late as she came to the hospital after 4 days. Her condition deteriorated rapidly as she developed septic shock and died within 2 days.

**Conclusions:**

Necrotizing fasciitis can be fatal when not recognized and without early intervention. Clinicians and surgeons alike should have a greater level of suspicion and appreciation for this uncommon yet lethal infection.

## Introduction

Necrotizing soft tissue infection (NSTI) targets skin, subcutaneous tissue, muscle or fascia and the infection may spread to involve adjacent tissue planes [1]. Classification systems vary based on tissue plane level or that of the microorganisms involved [2]. Based on the causative organism it is commonly categorized as type I or II necrotizing fasciitis (NF) [3] but some authors choose to extend this up to type III and IV when atypical organisms are included [4,5]. Multiple risk factors favor development of NF, which include loss of integrity of barrier mechanisms and conditions eventually predisposing to impaired immunity [2,6] among others. Few cases are diagnosed early due to the absence of specific symptoms; this predisposes to increased mortality because it can delay definitive surgical intervention and delay in diagnosis has been shown to be a major contributing factor for death [7]. NF is considered a rare and potentially fatal condition [4] and two cases are described here.

## Case presentation

### Case 1

**Figure 1:**
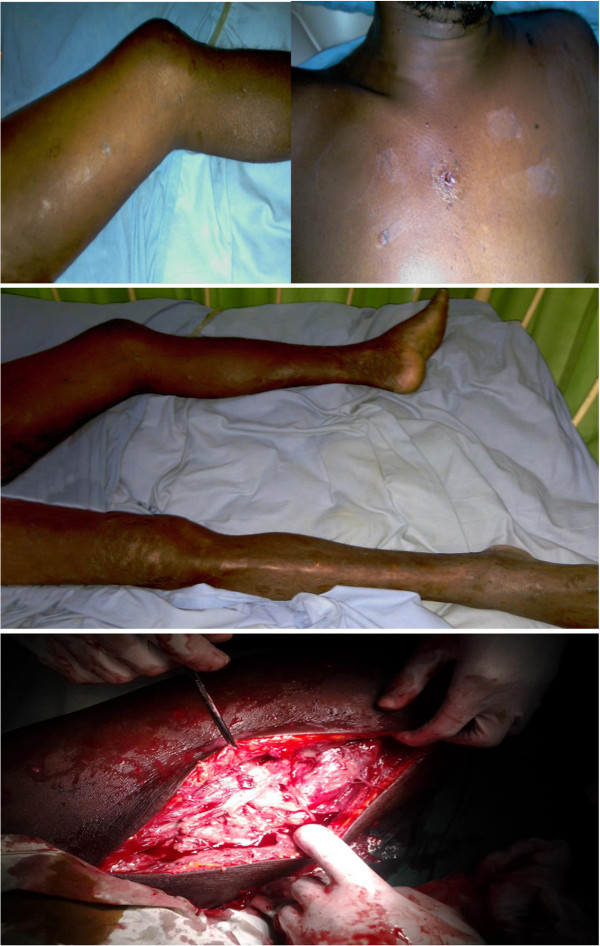
**Initial clinical presentation and surgical intervention.** The two top images depict the various healed and recent injuries sustained by the patient. The image in the middle shows a laterally rotated swollen left thigh and lower limb. The bottom image depicts necrotic muscle on surgical debridement of the left thigh.

A previously healthy 44-year-old South Asian man who worked as a groundskeeper and caretaker presented with left lower limb pain of 3 days’ duration with fever. The pain was significant enough to cause subjective weakness and his limb was found to be warm to touch and swollen. A preliminary clinical diagnosis of cellulitis was made and he was started on low-dose intravenous cloxacillin. The clinical situation worsened rapidly with progressive limb swelling and he became more septic. He developed absolute constipation and his abdominal girth increased during this timeframe and intestinal obstruction was suspected. He was transferred to our tertiary care center for further evaluation and management. An examination revealed multiple superficial wounds and healed injuries over his torso and legs (Figure 1). He appeared ill and was febrile. His left leg was noted to be kept in a laterally rotated position and the whole leg was swollen. It was also warm and tender to touch but did not exhibit any obvious superficial skin changes (Figure 1). There were crepitations on auscultation in both lung bases. His abdomen was distended but soft and percussion note was tympanic. Auscultation revealed diminished bowel sounds. Cardiovascular and neurological systems were normal on examination.

**Figure 2:**
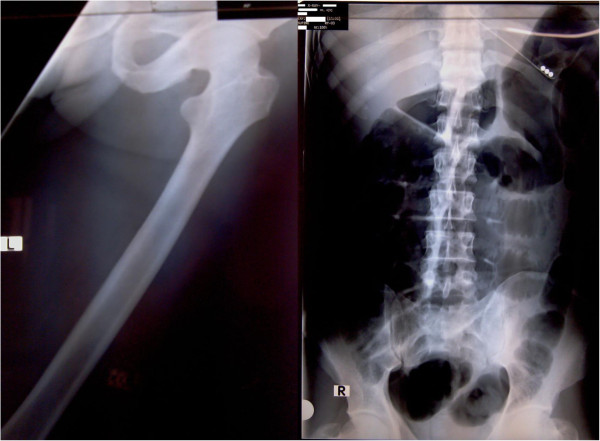
**X-ray imaging of left thigh and abdomen.** Image on the left is an X-ray of the left thigh; it failed to demonstrate presence of gas. Image on the right is an X-ray of the abdomen in supine position which demonstrates dilated large bowel loops favoring the clinical picture of intestinal obstruction.

**Figure 3:**
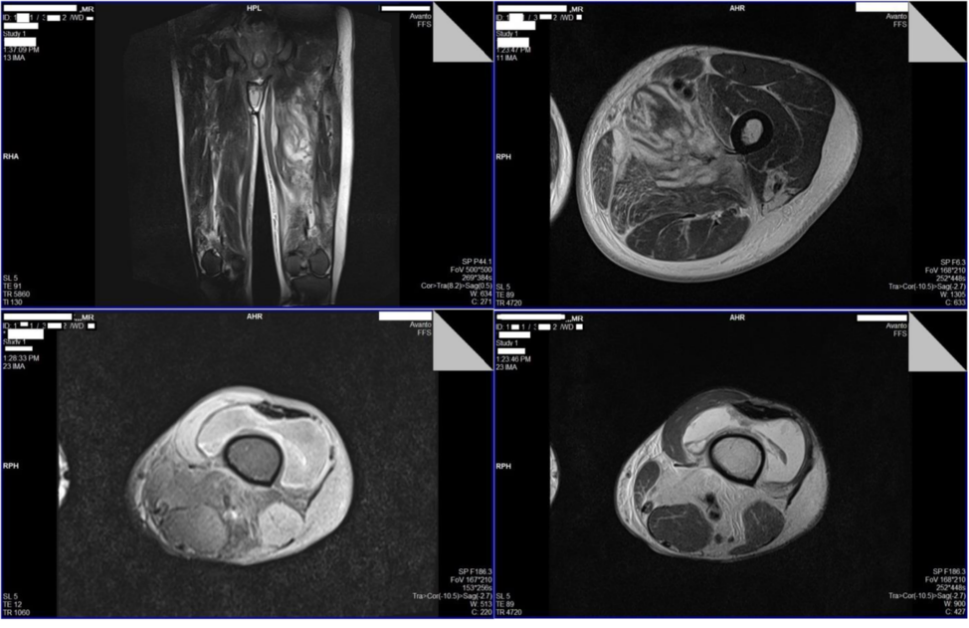
**Magnetic resonance imaging of the left lower limb.** Row 1: T2-weighted sagittal and axial view magnetic resonance imaging cuts of the left thigh demonstrate loss of normal architecture and edema of the muscle of the adductor compartment and numerous cystic areas. Row 2: Fat-suppressed T1-weighted image on the left, and T2-weighted image on the right, both axial magnetic resonance imaging cuts at the knee level demonstrate presence of gas with knee joint effusion.

**Figure 4:**
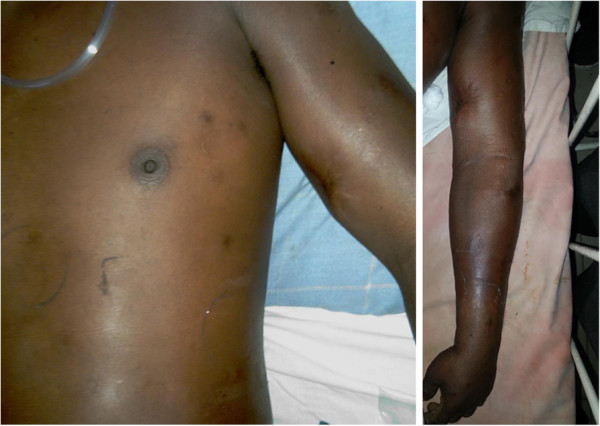
**Clinical deterioration and progression of necrotizing fasciitis.** Image depicts necrotizing fasciitis ascending to involve the chest and left upper limb.

Whole blood analysis revealed an elevated leucocyte count of 25.87 × 10^9^μ/L (normal: 4 to 10) which was predominantly neutrophilic (83%; normal: 50 to 70) with preserved hemoglobin and platelets. Inflammatory markers were elevated with an erythrocyte sedimentation rate (ESR) of 51mm for the first hour (normal: < 15). His renal functions were within reference range and remained normal throughout. An X-ray of his abdomen revealed dilated gaseous bowel loops (Figure 2) while an X-ray of his left thigh failed to demonstrate any abnormality or gas (Figure 2). An urgent magnetic resonance imaging (MRI) of his thigh revealed marked edema of the muscle of the adductor compartment of his left thigh with numerous cystic areas of peripheral enhancement and gas. Fascial involvement was seen extending to involve his hip and there was a concomitant large left-side knee joint effusion. Appearances were that of a NSTI with muscle and fascial involvement (Figure 3). Based on the clinical picture and MRI interpretation a diagnosis of NF was made. Surgical debridement was carried out urgently and intravenous cloxacillin was continued in high dosage together with intravenous metronidazole. During debridement, a copious quantity of necrotic material and fluid was cleared (Figure 1). Deep tissue culture taken at surgery revealed methicillin-resistant *Staphylococcus aureus* (MRSA) and intravenous vancomycin was added to the antibiotic regimen. Blood cultures were persistently negative. The intestinal obstruction resolved spontaneously with passage of stools and flatus. Although follow-through debridement and intervention were planned the patient became hemodynamically unstable and unsuitable for general anesthesia and induction as he remained inotrope dependent in septic shock. The unavoidable delay resulted in the infection rapidly spreading to involve his upper trunk and further ascending to involve his left upper limb as well over 48 hours (Figure 4). His clinical condition was reflected in our investigations because his leucocyte levels rose to 33.92 × 10^9^/L (normal: 4 to 10) although his blood cultures remained negative. He developed deteriorating liver functions with an elevated international normalized ratio of 1.43, an aspartate amino transferase of 97U/L (normal: 10 to 35) an alanine amino transferase of 74U/L (normal: 10 to 40) and an alkaline phosphatase value of 1091U/L (normal: 100 to 360). Arterial blood gas revealed a compensated metabolic acidosis, with a pH of 7.41, bicarbonate (HCO^3−^) of 17.1mmol/L (normal: 22 to 26) and a partial pressure of carbon dioxide of 27mmHg (normal: 35 to 45). However, his renal functions remained normal. His creatinine kinase was elevated with a value of 776U/L (normal: 25 to 174). Human immunodeficiency virus screening was negative as was hepatitis C antibodies. On day 7, he died.

### Case 2

**Figure 5:**
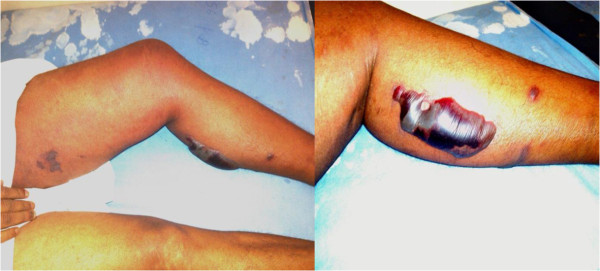
**Superficial skin manifestations of necrotizing fasciitis.** Image on the left shows early skin involvement of the left medial aspect of the thigh and bullae formation of the calf, which is more clearly demonstrated on the image on the right.

A 35-year-old South Asian woman who was previously diagnosed with systemic lupus erythematosus (SLE) and was on immunosuppressive therapy consisting of prednisolone and azathioprine presented with a 4-day history of fever and left lower limb pain. On examination she was febrile with a tender left leg was which was mildly erythematous and warm to touch and initially without any obvious skin manifestations such as bullae or blisters. Systemic examination was normal including preserved hemodynamic parameters. Blood cultures were taken and she was started on empirical antibiotics of intravenous cloxacillin. However, within 12 hours she developed bullae (Figure 5) on the medial aspect of her left calf and thigh. She deteriorated rapidly soon afterwards with hemodynamic compromise. Urgent debridement was undertaken.

Whole blood analysis demonstrated neutrophilic predominant (90%) leucopenia of 3.87 × 10^9^/L (4 to 11) and a mild anemia with hemoglobin of 9.9g/dL (normal: 11 to 18) which was normocytic and normochromic. Her preliminary renal profile was unaltered. Her international normalized ratio was mildly deranged at 1.42 (normal: 0.9 to 1.1), aspartate transaminase was elevated at 55U/L (normal: 10 to 35) but alkaline phosphatase and proteins were normal. Her ESR was elevated at 110mm for the first hour. Arterial blood gas demonstrated partially compensated metabolic acidosis with a pH of 7.35 (normal: 7.5 to 7.45) and a HCO^3−^ of 13.2mm/L (normal: 22 to 26). On admission she had normal serum sodium of 144mmol/L (normal: 135 to 148) and serum potassium of 3.8mmol/L (normal: 3.5 to 5.1) and a serum creatinine of 69μmol/L (normal: 60 to 120). Random blood sugar was 85mg/dL (normal: 79 to 140). Blood cultures and deep tissue cultures failed to reveal any organisms. Following surgery she remained in intensive care but she rapidly deteriorated and became inotrope dependent. She died 34 hours into hospital admission.

## Discussion

The first patient had NF and muscle necrosis of the adductor compartment of his thigh from the outset, but failure of the primary care center to realize the masked signs of NF led to the infection moving up to involve his upper trunk and arm. The delay in definitive management resulted in spread of infection with eventual sepsis and death. Case 2 was an immunocompromised patient who presented late. The inability of her body to mount an effective immune response resulted in rapid progression of NF with sepsis and septic shock despite urgent surgical and medical intervention.

Diagnosis of NF is a challenge to a clinician because it is a rare entity and there may be no obvious pointers favoring its diagnosis [8]. Expected manifestations like skin necrosis are not always obvious, and care should be taken to search for suggestive local (severe spontaneous pain that is disproportional to the degree of inflammation, indurated edema, bullae, cyanosis, skin pallor, absence of lymphangitis, skin hypoesthesia, crepitation, muscle weakness) and systemic signs of ongoing sepsis [2,6]. Furthermore the classic bronze or reddish discoloration of skin due to clostridial infections may not be commonly visualized due to the already tanned skin complexion of Asians, although Case 2 demonstrated typical skin manifestations with bullae formation. In Case 1 the possible missed sign was the out of proportion pain with a sense of weakness and heaviness which are nonspecific presenting features of gas-forming NF [9]. These were mistaken for a milder form of infection, cellulitis, with absence of superficial skin manifestations.

Risk factors include compromised integrity of skin or mucous membranes, diabetes, arteriopathy, alcoholism, obesity, immunosuppression, malnutrition, renal failure, and age > 60 years. Non-steroidal anti-inflammatory drugs have been suggested as possible risk factors for NF [2,6]. Both our patients had predisposing factors. The patient in Case 1 suffered multiple injuries secondary to his occupation as a laborer, but none were recent. However, *Clostridium* spores may remain dormant for many years before germination and resultant NF [10]. NF in SLE is uncommon [11] and Kamran *et al.* state that only 13 cases were reported up to 2008 [12]. A dampened immune system due to immunosuppressive (azathioprine with prednisolone in our patient) therapy, the disease process per se [12] or skin fragility secondary to prednisolone [13] could be additional predisposing factors for patients with SLE to get NF.

Although any part of the body may be involved, the lower limbs are the most commonly affected sites for infection (28%) [14]. The absence of fibrous attachments in the limbs and trunk lead to widespread infection and tissue destruction. Infection can also spread to venous and lymphatic channels with resultant edema and thrombosis of blood vessels which cause ischemia and gangrene of subcutaneous fat and dermis [2]. The involvement of the trunk carries a poorer prognosis compared with the extremities in isolation [15]. The rapidity at which the infection spread up the lower limbs in both our patients and the involvement of the trunk and upper limb in Case 1 can thus be explained.

Polymicrobial NF infections are poorly demonstrated on blood cultures which are found positive only in 20 to 27% [3,7] of patients. Neither of our patients’ blood cultures became positive. However, Case 1 had MRSA present on deep tissue culture. But the presence of gas in MRI suggested presence of an additional gas-forming organism, possibly a clostridial species. Although NF is commonly classified into type I and II, some extend the classification further and identify Gram negative or clostridial induced as type III and fungal-induced NF as type IV [4,5,16]. Type III due to clostridial species with muscle involvement is also considered clostridial myonecrosis. Clostridial sepsis can be secondary to trauma due to penetrating injuries, underlying intestinal pathology or even occur spontaneously. The patient in Case 1 most probably had type III NF as gas was present with muscle necrosis. In Case 2 neither blood nor deep tissue culture yielded growth, in keeping with culture patterns observed in polymicrobial NF or it may also be due to the immunocompromised state which may result in atypical organisms causing NF in SLE, for example *Pseudomonas aeruginosa*, and *Serratia marcescens*[17,18].

Imaging aids diagnosis. X-rays can show gas, although only in a minority (13%) of cases, and show increased soft tissue thickness. Ultrasound can help identify fascial edema and gas and fluid collection, having a sensitivity of 88.2% and a higher specificity of 93.3%, although user limitations may affect interpretation. Contrast-enhanced computed tomography can reveal soft tissue air and fluid and abscess collection, but its use may be limited by concomitant renal failure. MRI has been found to have a sensitivity of 100% and specificity of 86%. It can demonstrate gas bubbles as signal voids, and identify fascial fluid secondary to necrosis and inflammatory edema because it causes variation in signal intensity. When not enhanced the severity may be overestimated due to its inability to differentiate affected tissue from that of non-affected, and underestimated when gadolinium enhanced because tissue hypoperfusion may limit uptake [19-22]. Overall, MRI is considered the investigation of choice, but none of the imaging modalities should delay definitive surgical intervention [23]. Case 1 demonstrated typical imaging changes on MRI with presence of gas with fascial and adjacent muscle compartment involvement as NSTI has been known to cross and involve neighboring tissue planes [1].

A Laboratory Risk Indicator for Necrotizing Fasciitis (LRINEC) score of 6 or above (parameters made of total white cell count, hemoglobin, sodium, glucose, serum creatinine, and C-reactive protein) introduced by Wong *et al.* in their retrospective analysis [24] was found to be useful in detecting NF early. Although our patients’ ESR was elevated and available it is not included in the scoring system and, unfortunately, C-reactive protein is not available in the free health-care system offered in our country, thus limiting initial assessment using the LRINEC scoring system, but clinical sense should take precedence when other parameters are unavailable and when the LRINEC score contradicts diagnosis of NF on clinical grounds [25].

Treatment is mainly surgical with relevant early radical debridement of devitalized tissue. Being too judicious and attempting to conserve tissue may in fact be detrimental in the long run as it has been shown to worsen mortality [26]. In addition, supportive measures should be implemented with broad spectrum antibiotic treatment to target the spectrum of causative microorganisms (*Streptococcus pyogenes*, *Staphylococcus aureus* including MRSA, and Gram-negative aerobes and anaerobes) until cultures are available [2]. Other novel treatment options and adjuncts have been tried and suggested, for example intravenous immunoglobulin to counteract systemic toxicity produced by beta-hemolytic Streptococci [27] and hyperbaric oxygen as an effective adjunct in reducing morbidity and mortality [28], although it remains disputed [29]. Vacuum-assisted closing as a postsurgical adjunct to expedite healing [30] also has been tried.

NF without treatment has a mortality of 100% [2] but with medical and mainly surgical intervention it now has an overall mortality of 16 to 20% [15,31]. Type I NF was found to have a mortality of 21% by Wong *et al.*[7] but mortality in type III NF due to clostridial species can range from 25 to 80% [32]. Female gender, presence of malignant disease, and diabetes mellitus were found to be independent factors associated with increased mortality in the idiopathic variants [33]. However time plays the most significant role as a delay of no greater than 24 hours can literally double the mortality rate [27]. The primary focus in the first patient was shifted to the intestinal obstruction which was due to two possible mechanisms. One was that he developed paralytic ileus, an observed complication of an abdominal abscess [34] which could occur with infection tracking up to involve the psoas. The second possibility is of an occult intestinal malignancy, which is known to have an association with type III NF due to *Clostridium* species [35,36], which in turn could track through fascial and muscle planes to involve the lower limb and upper trunk. However, since lower limb features were predominant in the absence of any abdominal involvement at the outset and the transient intestinal obstruction resolved, the former is more likely. The urgency of time in managing NF is reinforced as there is little doubt that mortality was due to failure in identifying and curtailing the disease in time in Case 1 and delay in seeking management in Case 2.

## Conclusions

NSTIs are a poorly recognized group of lethal conditions. Clinicians and surgeons should have a high index of suspicion when symptomology is out of proportion to the clinical presentation. Failing to identify classical risk factors and not clinching the diagnosis early, non-aggressive treatment and delayed definitive surgical intervention favor mortality.

## Consent

Written informed consent was obtained from the patients’ next of kin for the publication of this case report and accompanying images. Copies of the written consent are available for review by the Editor-in-Chief of this journal.

## Abbreviations

ESR: Erythrocyte sedimentation rate; HCO^3−^: Bicarbonate; LRINEC: Laboratory Risk Indicator for Necrotizing Fasciitis; MRI: Magnetic resonance imaging; MRSA: Methicillin-resistant *Staphylococcus aureus*; NF: Necrotizing fasciitis; NSTI: Necrotizing soft tissue infection; SLE: Systemic lupus erythematosus.

## Competing interests

The authors declare that they have no competing interests.

## Authors’ contributions

AK, MRN, JY, and TK diagnosed the clinical scenario. MRN and AK researched and drafted the document. All authors provided care for the patient. All authors read and approved the final manuscript.

## Authors’ information

MRN is a registrar of medicine at the National Hospital of Sri Lanka, Colombo. JY is a senior registrar in medicine at the National Hospital of Sri Lanka, Colombo. TK is a senior registrar in medicine at the National Hospital of Sri Lanka, Colombo. AK is a consultant physician in acute medicine at the National Hospital of Sri Lanka, Colombo.

## References

[B1] Smith GH Huntley JS Keenan GF Necrotising myositis: a surgical emergency that may have minimal changes in the skin Emerg Med J 2007 24 e8 10.1136/emj.2006.041723 PMC2658222 17251603

[B2] Puvanendran R Huey JC Pasupathy S Necrotizing fasciitis Can Fam Physician 2009 55 981 987 PMC2762295 19826154

[B3] Elliott D Kufera JA Myers RA The microbiology of necrotizing soft tissue infections Am J Surg 2000 179 361 366 10.1016/s0002-9610(00)00360-3 10930480

[B4] Davoudian P Flint NJ Necrotizing fasciitis 2012 Critical Care & Pain: Continuing Education in Anaesthesia doi:10.1093/bjaceaccp/mks033

[B5] Necrotizing Fasciitis http://www.nycpm.edu/surgclub/necrotizing.pdf

[B6] Roujeau JC Necrotizing fasciitis. Clinical criteria and risk factors Ann Dermatol Venereol 2001 128 376 381 11319368

[B7] Wong CH Chang HC Pasupathy S Khin LW Tan JL Low CO Necrotizing fasciitis: clinical presentation, microbiology, and determinants of mortality J Bone Joint Surg Am 2003 85-A 1454 1460 12925624

[B8] Anaya DA Dellinger EP Necrotizing soft-tissue infection: diagnosis and management Clin Infect Dis 2007 44 705 710 10.1086/511638 17278065

[B9] Wiersema BM Scheid DK Psaradellis T A rare trifocal presentation of *Clostridium septicum* myonecrosis Orthopedics 2008 31 274 10.3928/01477447-20080301-43 19292235

[B10] Clostridial myonecrosis http://www.uptodate.com/contents/clostridial-myonecrosis

[B11] Mendez EA Espinoza LM Harris M Angulo J Sanders CV Espinoza LR Systemic lupus erythematosus complicated by necrotizing fasciitis Lupus 1999 8 157 159 10.1191/096120399678847452 10192511

[B12] Kamran M Wachs J Putterman C Necrotizing fasciitis in systemic lupus erythematosus Semin Arthritis Rheum 2008 37 236 242 10.1016/j.semarthrit.2007.04.005 17570472

[B13] Hashimoto N Sugiyama H Asagoe K Hara K Yamasaki O Yamasaki Y Makino H Fulminant necrotising fasciitis developing during long term corticosteroid treatment of systemic lupus erythematosus Ann Rheum Dis 2002 61 848 849 10.1136/ard.61.9.848 PMC1754225 12176816

[B14] Golger A Ching S Goldsmith CH Pennie RA Bain JR Mortality in patients with necrotizing fasciitis Plast Reconstr Surg 2007 119 1803 1807 10.1097/01.prs.0000259040.71478.27 17440360

[B15] Serinken M Erdur B Sener S Kabay B Cevik A A Case of Mortal Necrotizing Fasciitis of the Trunk Resulting From a Centipede (*Scolopendra moritans*) Bite Internet J Emerg Med 2004 2 2

[B16] Necrotising fasciitis http://www.dermnetnz.org/bacterial/necrotising-fasciitis.html

[B17] Huang JW Fang CT Hung KY Hsueh PR Chang SC Tsai TJ Necrotizing fasciitis caused by *Serratia marcescens* in two patients receiving corticosteroid therapy J Formos Med Assoc 1999 98 851 854 10634026

[B18] Nimesh KP Laura M A Rare Cause of Necrotizing Fasciitis in a Patient With Systemic Lupus Erythematosus C56 PULMONARY AND NON-PULMONARY CRITICAL CARE: GREAT CASES!: American Thoracic Society: A4596: American Thoracic Society International Conference Abstracts

[B19] Fugitt JB Puckett ML Quigley MM Kerr SM Necrotizing fasciitis Radiographics 2004 24 1472 1476 10.1148/rg.245035169 15371620

[B20] Angoules AG Kontakis G Drakoulakis E Vrentzos G Granick MS Giannoudis PV Necrotising fasciitis of upper and lower limb: a systematic review Injury 2007 38 Suppl 5 S19 S26 10.1016/j.injury.2007.10.030 18048033

[B21] Yen ZS Wang HP Ma HM Chen SC Chen WJ Ultrasonographic screening of clinically-suspected necrotizing fasciitis Acad Emerg Med 2002 9 1448 1451 10.1111/j.1553-2712.2002.tb01619.x 12460854

[B22] Schmid MR Kossmann T Duewell S Differentiation of necrotizing fasciitis and cellulitis using MR imaging AJR Am J Roentgenol 1998 170 615 620 10.2214/ajr.170.3.9490940 9490940

[B23] Stoneback JW Hak DJ Diagnosis and management of necrotizing fasciitis Orthopedics 2011 34 196 10.3928/01477447-20110124-20 21410101

[B24] Wong CH Khin LW Heng KS Tan KC Low CO The LRINEC (Laboratory Risk Indicator for Necrotizing Fasciitis) score: a tool for distinguishing necrotizing fasciitis from other soft tissue infections Crit Care Med 2004 32 1535 1541 10.1097/01.ccm.0000129486.35458.7d 15241098

[B25] Wilson MP Schneir AB A case of necrotizing fasciitis with a LRINEC score of zero: clinical suspicion should trump scoring systems J Emerg Med 2013 44 928 931 10.1016/j.jemermed.2012.09.039 23287745

[B26] Freischlag JA Ajalat G Busuttil RW Treatment of necrotizing soft tissue infections. The need for a new approach Am J Surg 1985 149 751 755 10.1016/s0002-9610(85)80180-x 4014552

[B27] Seal DV Necrotizing fasciitis Curr Opin Infect Dis 2001 14 127 132 10.1097/00001432-200104000-00003 11979121

[B28] Riseman JA Zamboni WA Curtis A Graham DR Konrad HR Ross DS Hyperbaric oxygen therapy for necrotizing fasciitis reduces mortality and the need for debridements Surgery 1990 108 847 850 2237764

[B29] Hassan Z Mullins RF Friedman BC Shaver JR Brandigi C Alam B Mian MA Treating necrotizing fasciitis with or without hyperbaric oxygen therapy Undersea Hyperb Med 2010 37 115 123 20462144

[B30] Al-Subhi F Zuker R Cole W Vacuum-assisted closure as a surgical assistant in life-threatening necrotizing fasciitis in children Can J Plast Surg 2010 18 139 142 10.1177/229255031001800412 PMC3006112 22131841

[B31] Frazee BW Fee C Lynn J Wang R Bostrom A Hargis C Moore P Community-acquired necrotizing soft tissue infections: a review of 122 cases presenting to a single emergency department over 12 years J Emerg Med 2008 34 139 146 10.1016/j.jemermed.2007.03.041 17976799

[B32] Bretzke ML Bubrick MP Hitchcock CR Diffuse spreading *Clostridium septicum* infection, malignant disease and immune suppression Surg Gynecol Obstet 1988 166 197 199 3344448

[B33] Taviloglu K Cabioglu N Cagatay A Yanar H Ertekin C Baspinar I Ozsut H Guloglu R Idiopathic necrotizing fasciitis: risk factors and strategies for management Am Surg 2005 71 315 320 15943405

[B34] Management of intra-abdominal abscesses http://www.ncbi.nlm.nih.gov/books/NBK6937/

[B35] Gibson MA Avgerinos DV Llaguna OH Sheth ND Myonecrosis secondary to *Clostridium septicum* in a patient with occult colon malignancy: a case report Cases J 2008 1 300 10.1186/1757-1626-1-300 PMC2588582 18992141

[B36] Larson CM Bubrick MP Jacobs DM West MA Malignancy, mortality, and medicosurgical management of *Clostridium septicum* infection Surgery 1995 118 592 597 discussion 597–598 10.1016/s0039-6060(05)80023-6 7570310

